# Supplementary material for: Impact of carbon dioxide removal technologies on deep decarbonization of the electric power sector
Source: Nat Commun. 2021 Jun 17;12:3732. doi: 10.1038/s41467-021-23554-6 (PMC8211809; doi:10.1038/s41467-021-23554-6)
Supplement: Supplementary file 1 — Supplementary Information [file 41467_2021_23554_MOESM1_ESM.pdf]

Impact of Carbon Dioxide Removal Technologies on Deep Decarbonization of the Electric Power Sector

John E.T. Bistline[1]*, Geoffrey J. Blanford[1]

Supplementary Note 1: Model Detail and Assumptions

EPRI's U.S. Regional Economy, Greenhouse Gas, and Energy (REGEN) model finds intertemporally cost-optimal electric sector pathways given assumptions about policies, technologies, and markets. Many capacity planning models (and nearly all integrated assessment models) do not adequately capture the spatial and temporal complexity of high renewable and very low CO_2 systems [1, 2, 3]. REGEN's large geographical scope and hourly temporal resolution allow the model to evaluate how the costs of electric sector technologies compare with their value, which can change in different locations, times, and deployment levels and can be difficult to capture in models with lower temporal resolution [4].

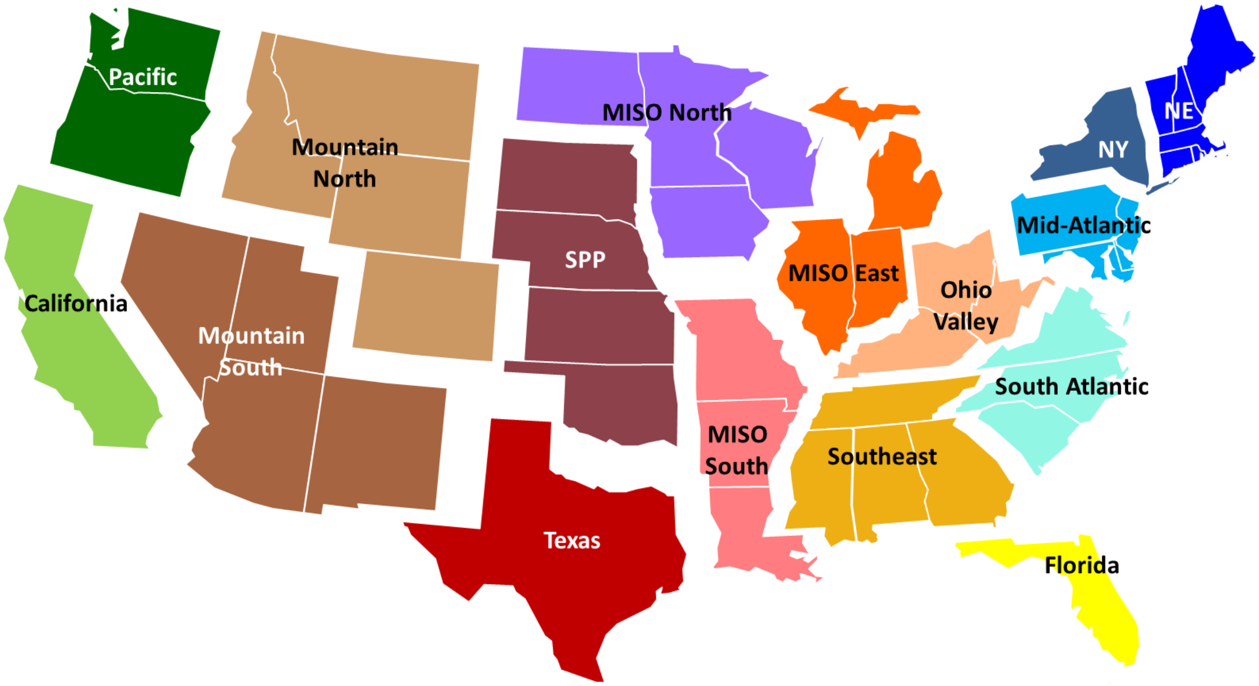

Supplementary Figure 1. Regional aggregation of REGEN for this study. The Lower 48 U.S. states are grouped into the 16 regions above. Source data are provided as a Source Data file.

Regional biomass supply feedstocks are shown in Supplementary Supplementary Figure **2** and are derived from the Forest and Agriculture Sector Optimization Model with Greenhouse Gases (FASOM-GHG). Additional detail on the forestry and agricultural biomass supply modeling is provided in Appendix B of the detailed REGEN documentation [4]. Note that the high fixed O&M costs for BECCS in Table 1 (main text) are due to the materials handling requirements for biomass; to the maintenance costs for the

[1] Electric Power Research Institute, 3420 Hillview Avenue, Palo Alto, CA 94304. Corresponding author. Email: jbistline@epri.com. Phone: 650-855-8517.

crushers, shredders, and pulverizers; and to diseconomies of scale if future plants are the size of current biomass combustion plants.

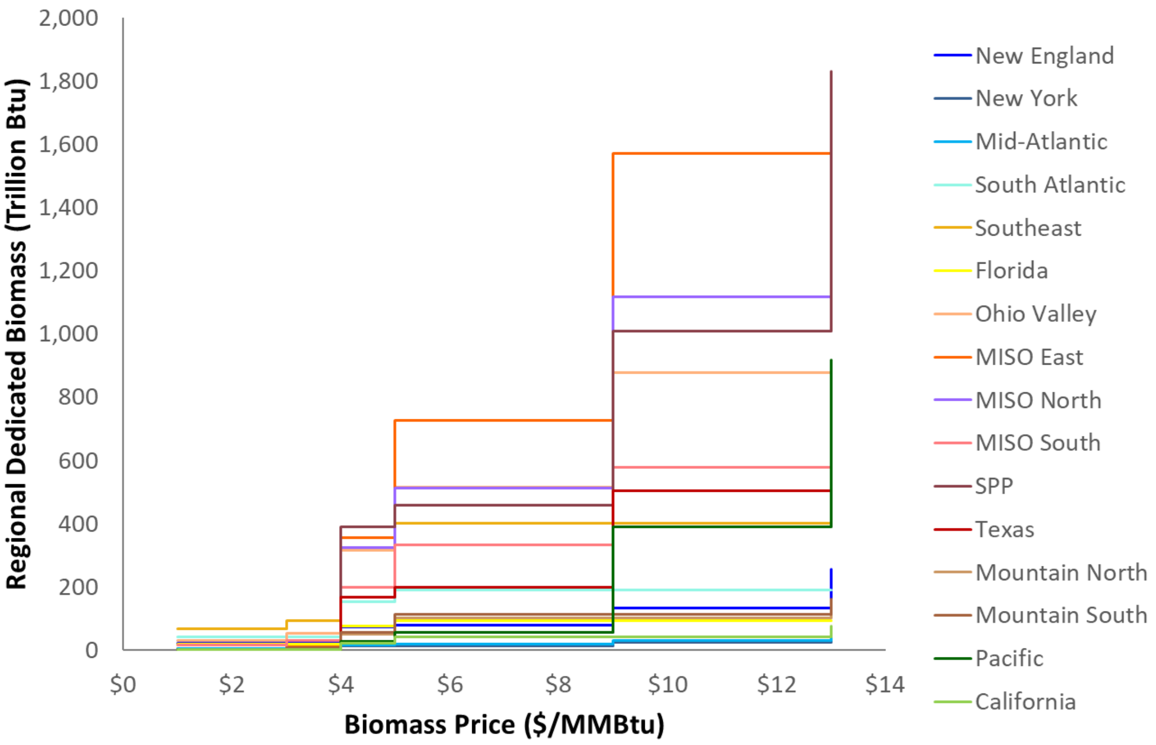

Supplementary Figure 2. Regional power sector biomass supply curves. Delivered biomass costs for electricity production are based on the Forest and Agriculture Sector Optimization Model with Greenhouse Gases (FASOM-GHG). State mappings are shown in Supplementary Figure 1. Source data are provided as a Source Data file.

The modeling incorporates two configurations for CCS-equipped natural gas capacity: natural gas combined cycle (NGCC) with post-combustion capture and oxy-combustion with an advanced power cycle. Technological cost and performance assumptions are based on Johnson and Swisher [5] and are summarized in Supplementary Table 1. Given these parameters, the scenarios in this analysis that include CCS-equipped gas in the least-cost mix generally select the oxy-combustion configuration with a higher capture rate.

Supplementary Table 1. Assumptions for carbon-capture-equipped natural gas technologies. Assumptions are based on Johnson and Swisher [5]. All values are expressed in 2018 U.S. dollars and are for nth-of-a-kind units.

	**Post-Combustion**	**Oxy-Combustion**
**Capital Cost**	$2,152/kW	$2,302/kW
**Investment Lifetime**	50 years	50 years
**Capture Rate**	90%	97%
**Heat Rate**	7.4 MMBtu/MWh	7.4 MMBtu/MWh
**Fixed O&M Cost**	$47/kW-yr	$44/kW-yr
**Variable O&M Cost**	$8.10/MWh (excluding fuel)	$3.14/MWh (excluding fuel)

Supplementary Figure 3 compares the "Reference" and "Breakthrough" battery costs with 2018 U.S. averages and values from NREL's Annual Technology Baseline (ATB) 2020.

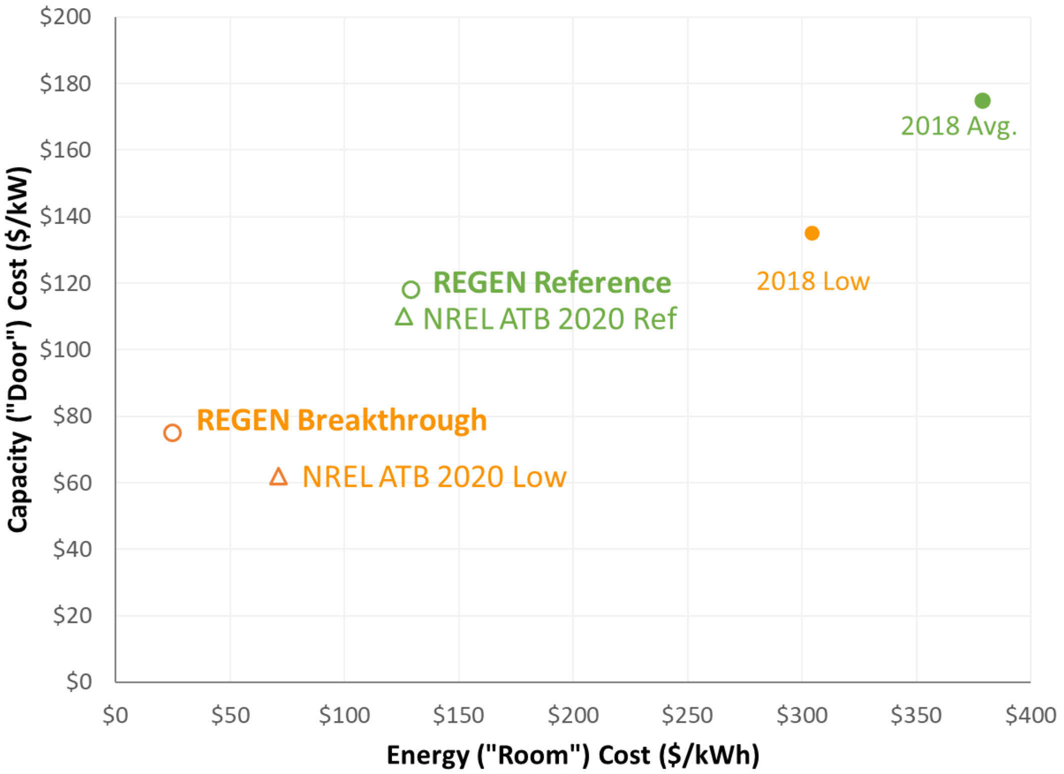

Supplementary Figure 3. Battery storage energy and capacity costs in the "Reference" and "Breakthrough" scenarios. Reference costs are based on EPRI [6]. Costs from NREL's Annual Technology Baseline (ATB) 2020 are also shown. Source data are provided as a Source Data file.

For hydrogen pathways, the model independently optimizes the capacity of hydrogen production via electrolysis, hydrogen storage, and generation from hydrogen turbines. The assumed electrolysis capital costs of $200/kW are at the lower range of current estimates (Supplementary Figure 4), though there is considerably uncertainty in the literature about current and future costs. Note that the costs at the bottom of the range come from BloombergNEF [7] projections for alkaline electrolysis systems, which assumes that costs for Western manufacturers approach levels of Chinese-made electrolyzers as production achieves greater scale. The cost of electricity is endogenously determined from the grid mix in the model.

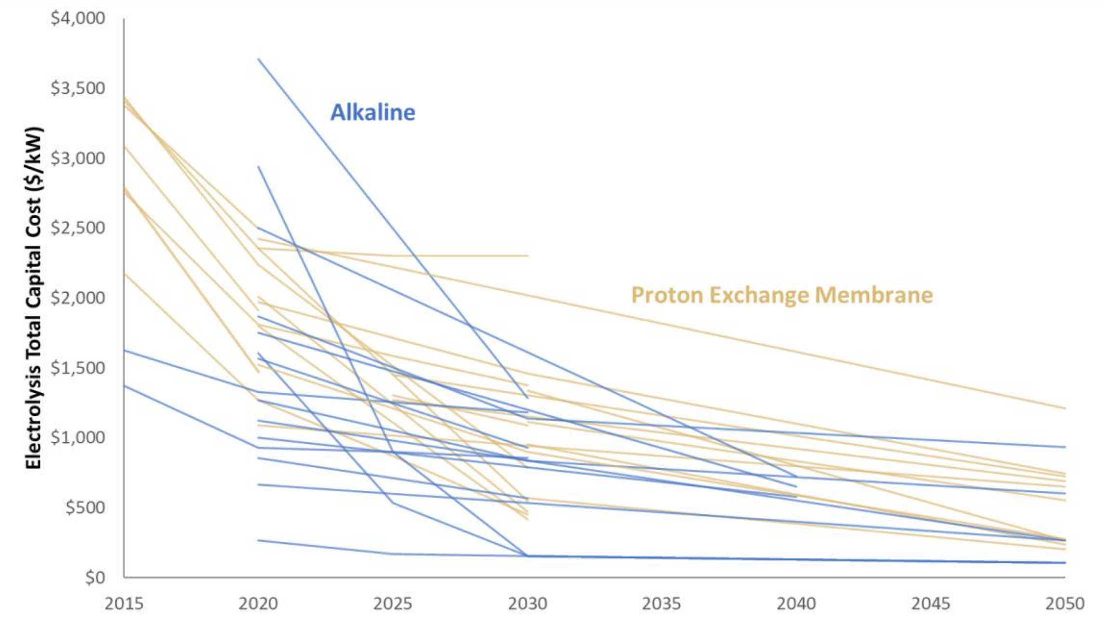

Supplementary Figure 4. Electrolysis system capital costs alkaline (blue) and proton exchange membrane (gold) technologies in the current literature. Cost projections come from a range of journal articles and reports. Source data and references are provided as a Source Data file.

REGEN models transport of captured CO_2 to injection sites where it is stored in saline aquifers (Supplementary Figure 5). Additional information about the CO_2 transport/storage formulation and assumptions in REGEN can be found in the detailed model documentation [4].

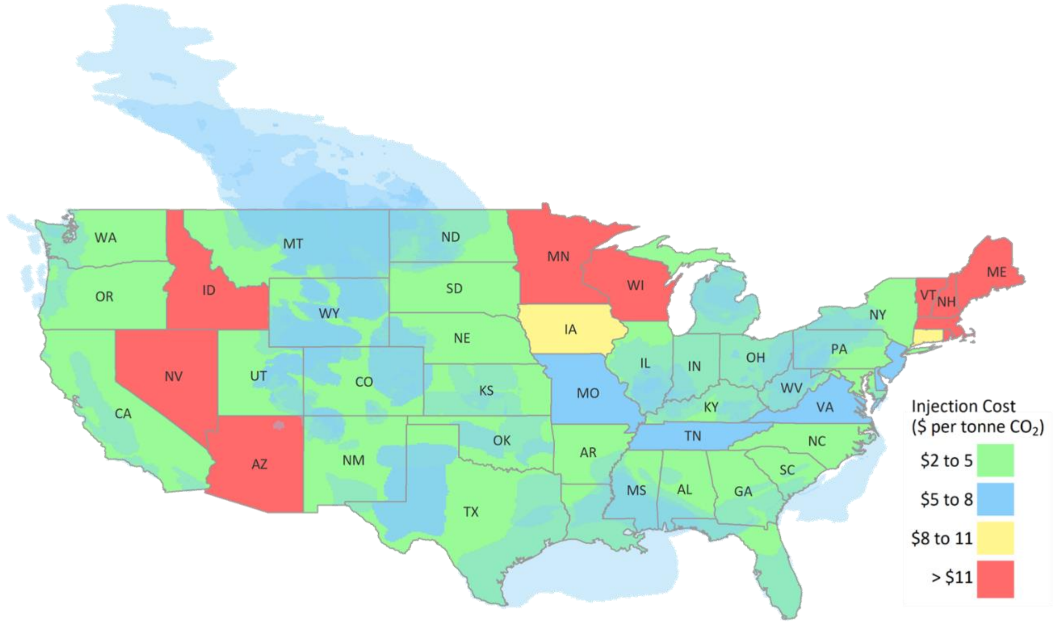

Supplementary Figure 5. Average CO_2 transport and storage costs by state. Values include inter-regional transport for states without storage capacity. Blue shading shows locations of saline aquifers. Source: EPRI [4].

Time-synchronized hourly load comes from the REGEN end-use model, which characterizes the economic and behavioral incentives for end-use technology adoption and captures heterogeneity across households, industries, and regions [4, 8]. The end-use model scenario is a dynamic model run where we use the 2050 hourly load profile results to describe electricity demand for the scenarios run with the single-year (static) electric sector model. To reflect the deep decarbonization context of the power sector sensitivities, the end-use model scenario assumes federal CO_2 pricing of \$50/t-CO_2 in all sectors and regions beginning in 2020, escalating at the model's discount rate of seven percent per year. The scenario also includes accelerated capital stock turnover for heavy-duty vehicles and industry. These assumptions give rise to extensive electrification with 67% load growth national between 2015 and 2050. Supplementary Figure 6 illustrates hourly load shape changes for California. The same regional load profiles are used for all electric sector scenarios.

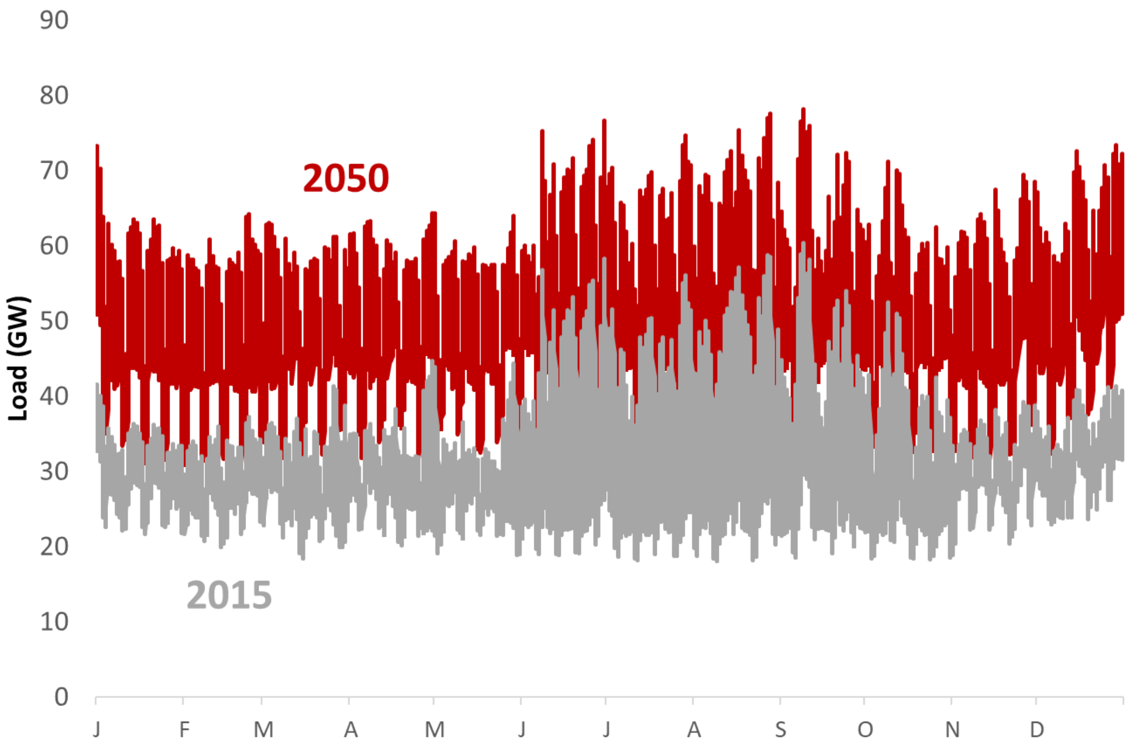

Supplementary Figure 6. Hourly load shape from California in 2015 and 2050. Profiles are outputs from the REGEN end-use model [4]. Source data are provided as a Source Data file.

Supplementary Figure 7 shows non-electric CO_2 emissions by sector for this deep decarbonization scenario. Gross CO_2 emissions decline 71% by 2050 relative to 2005 levels, and residual emissions come from hard-to-decarbonize end uses such as aviation and high-temperature industrial processes. The negative emissions from DAC and BECCS in the "140% Cap" scenario leads to approximately net-zero economy-wide CO_2 emissions by 2050 in this scenario.

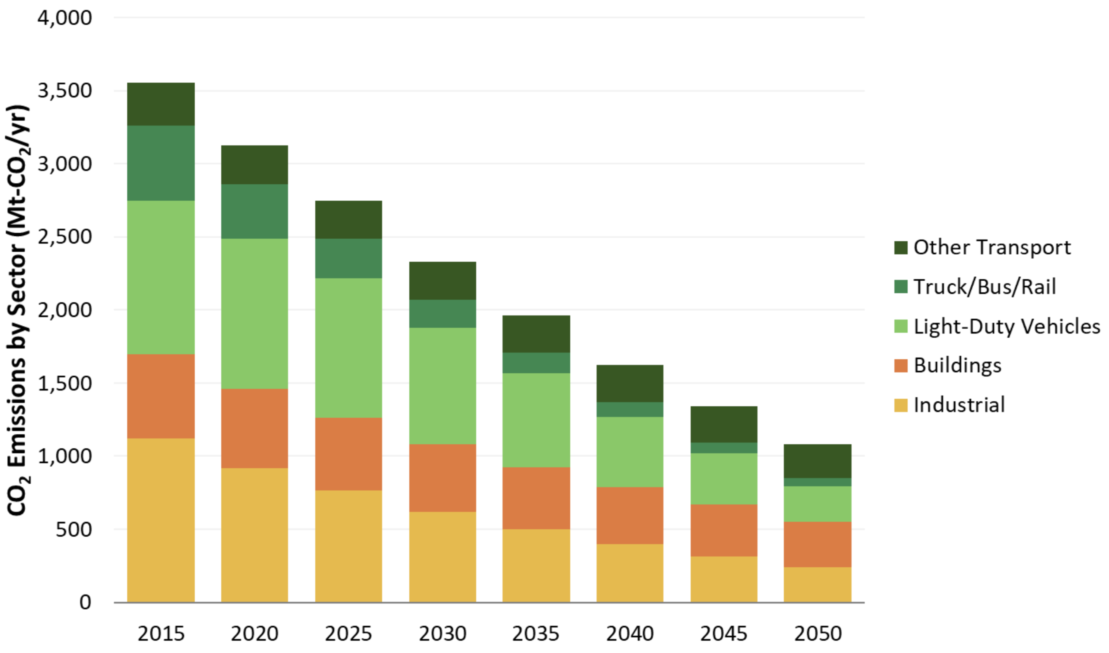

Supplementary Figure 7. CO_2 emissions by sector over time. Results are outputs from the REGEN end-use model [4]. Source data are provided as a Source Data file.

Supplementary Note 2: Additional Results

2.1. Investment and Generation Results

Supplementary Figure 8 shows generation and capacity across different CO_2 cap scenarios without CDR technologies. Least-cost strategies to 80% reductions continue current trends of expanding solar, wind, and battery storage and maintaining hydro, nuclear, and gas capacity. For deeper decarbonization targets, dispatchable very-low-CO_2 options play an important role. Advanced nuclear and hydrogen turbines are deployed here given their costs and value (117 and 612 GW in 100% cap scenario, respectively), but least-cost portfolios are region-specific (Supplementary Figure 10 and Supplementary Figure 11) and are sensitive to assumptions about the cost and performance of nascent technologies [9]. Wind and solar shares generally plateau around 80% generation shares, but saturation levels vary by region and policy target. Gas generation falls faster than capacity, and the presence of many lower utilization options near 100% decarbonization increases marginal abatement costs (Supplementary Figure 15).

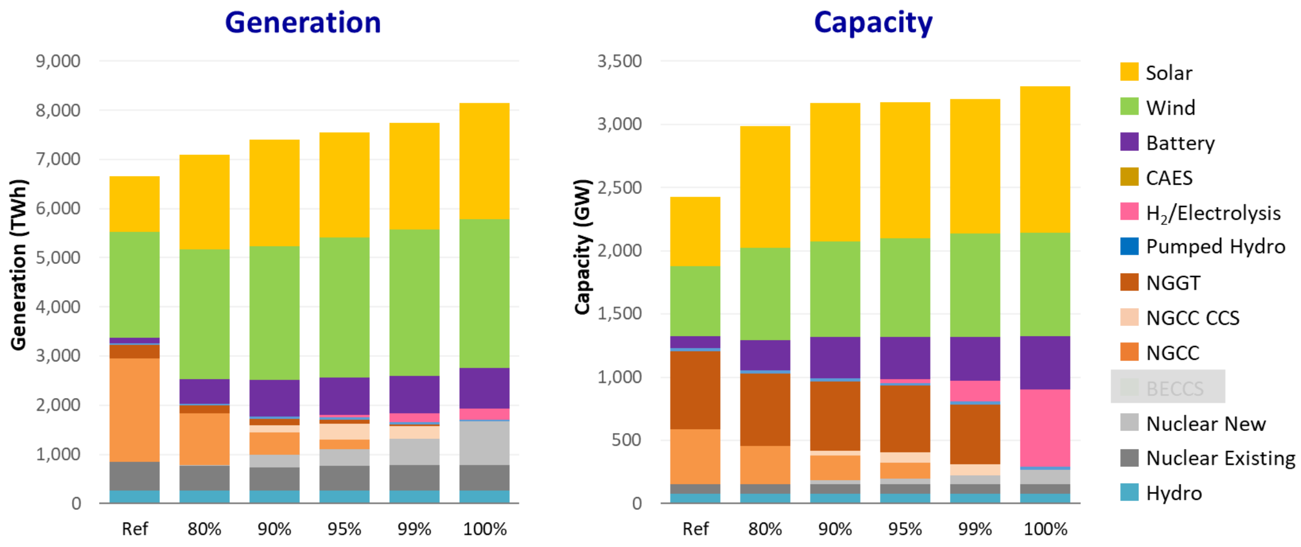

Supplementary Figure 8. National generation (left) and capacity (right) by technology and electric sector CO_2 cap scenario (% reduction from 2005 levels). Scenarios without CDR technologies are shown. Source data are provided as a Source Data file.

Supplementary Figure 9 shows changes to the generation and capacity mix when CDR is available relative to the scenarios without CDR technologies.

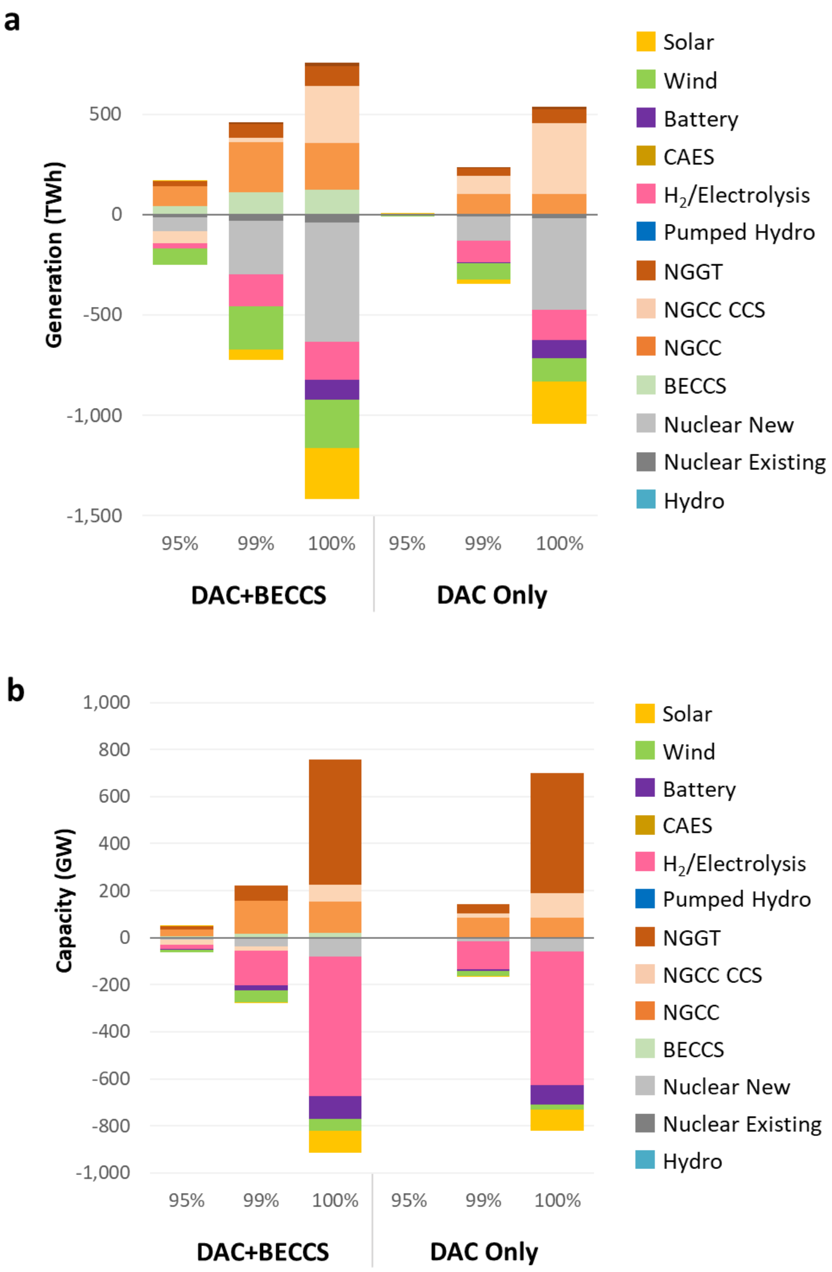

Supplementary Figure 9. Differences in generation (a, top panel) and capacity (b, bottom panel) relative to the scenario without CDR by technology and scenario. "Cap" refers to the electric sector CO_2 cap policy in terms of percentage reductions relative to 2005 levels. Positive (negative) values indicate increases (decreases) in the scenario with CDR availability relative to one without CDR. Source data are provided as a Source Data file.

The competitiveness of renewables and other low-carbon resources varies by region and scenario. Wind and solar shares generally rise with more stringent CO_2 limits (Supplementary Figure 10).

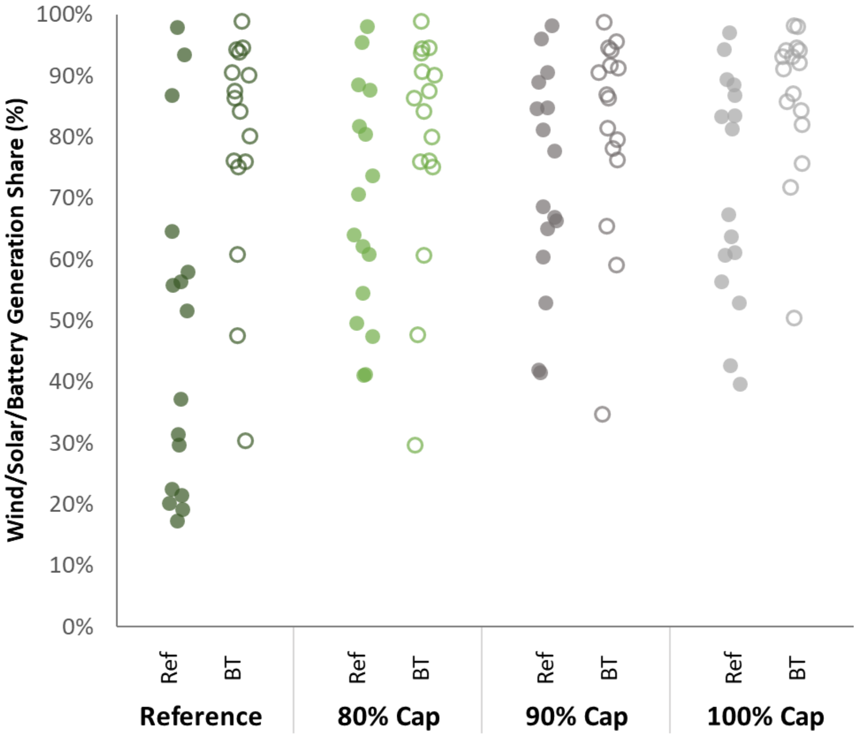

Supplementary Figure 10. Share of wind, solar, and battery generation (% in-region generation) for different model regions (dots), grouped by policy and technology scenario. "Cap" refers to the electric sector CO_2 cap policy in terms of percentage reductions relative to 2005 levels. Technology scenarios assume reference ("Ref") or breakthrough ("BT") renewables and energy storage costs. Source data are provided as a Source Data file.

Least-cost decarbonization pathways differ by region and policy stringency. Supplementary Figure 11 presents regional generation shares under the 100% cap scenario without CDR. Regions with lower-quality renewable resources in the Eastern U.S. deploy larger amounts of nuclear. Models without regional resolution (e.g., global integrated assessment models that may only represent the U.S. or North America as a single region) understate cost and value heterogeneity.

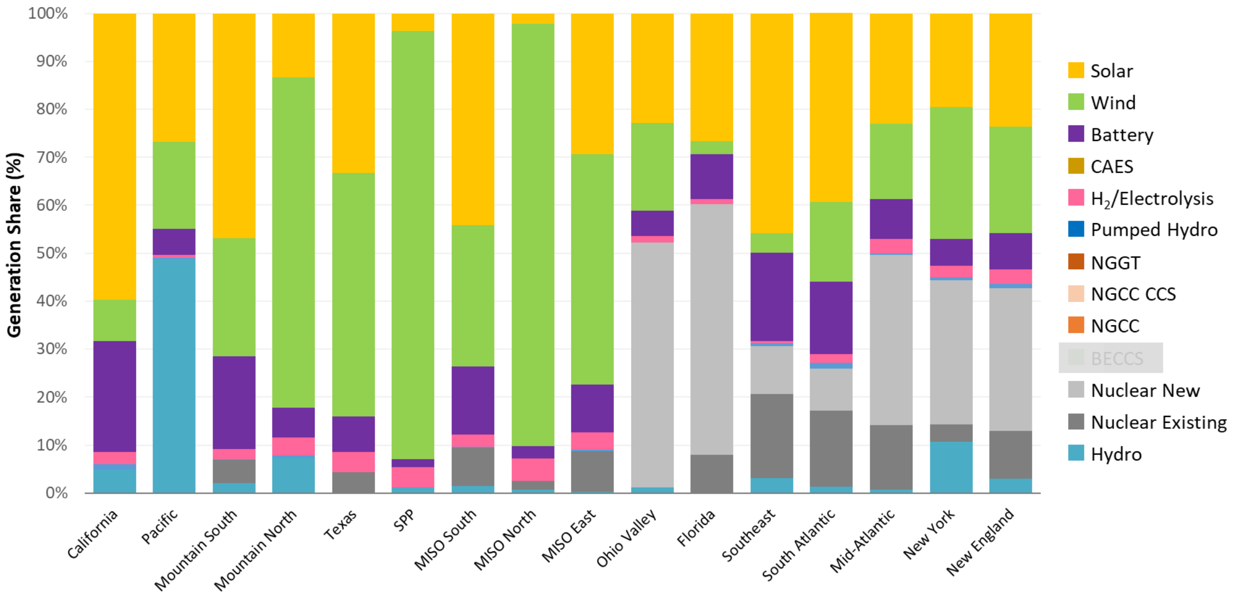

Supplementary Figure 11. Generation shares (% in-region generation by technology) by region for the 100% electric sector CO_2 cap scenario without CDR. Regional definitions are shown in Supplementary Figure **1**. Source data are provided as a Source Data file.

If the choice set of technologies to meet the net-zero policy target is restricted to renewables only, the least-cost mix relies most heavily on both diurnal and seasonal storage to manage synoptic and seasonal variability (Supplementary Figure 12). The increases in installed capacity, energy storage, and transmission lead incremental policy costs to increase 45% relative to the technology-neutral 100% cap.

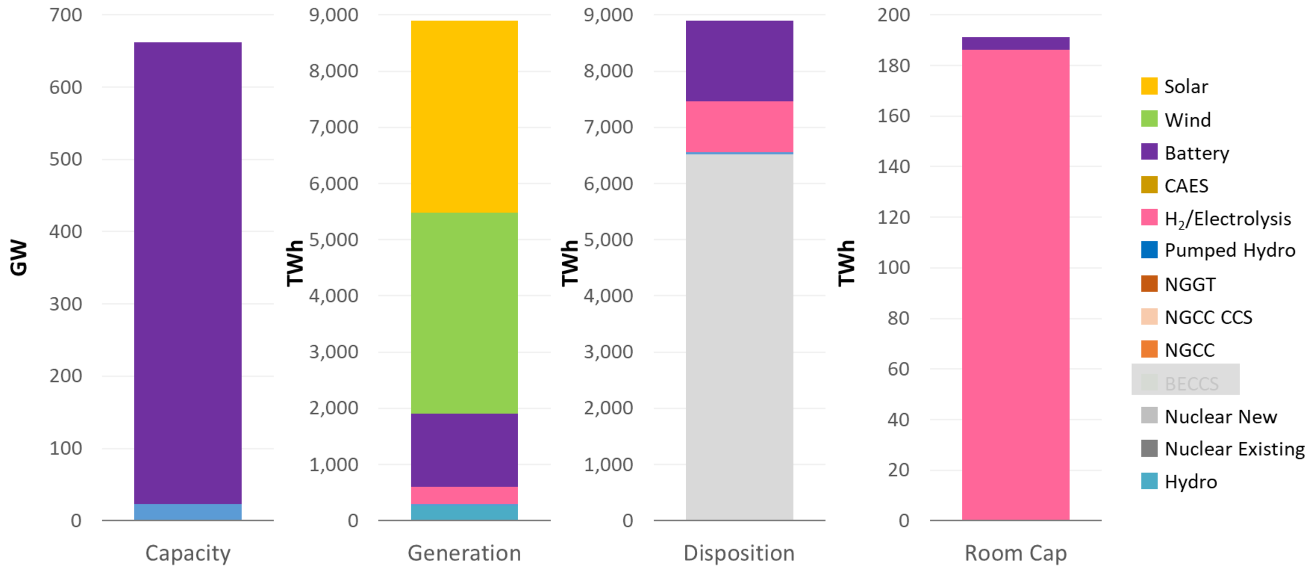

Supplementary Figure 12. National capacity, generation, disposition, and room cap for the 100% electric sector CO_2 cap scenario with renewables only. Reference technology costs are used. Source data are provided as a Source Data file.

With CO_2 caps beyond 80%, the extent of storage deployment increases, and the composition of the storage portfolio shifts toward longer-duration options (Supplementary Figure 13). Battery storage deployment is extensive in all scenarios, and cost reductions can increase capacity installations nearly eightfold in the reference case. Longer-duration energy storage technologies typically have low utilizations and higher capital costs, leading to lower marginal value per unit of capacity and making their economics challenging unless very high renewables or deep decarbonization policies are in place [10, 11]. These factors contribute to the high cost of these systems and their displacement when CDR is available. Note how the cost-minimizing mixes typically entail higher hydrogen turbine output relative to the sizing of the electrolysis input.

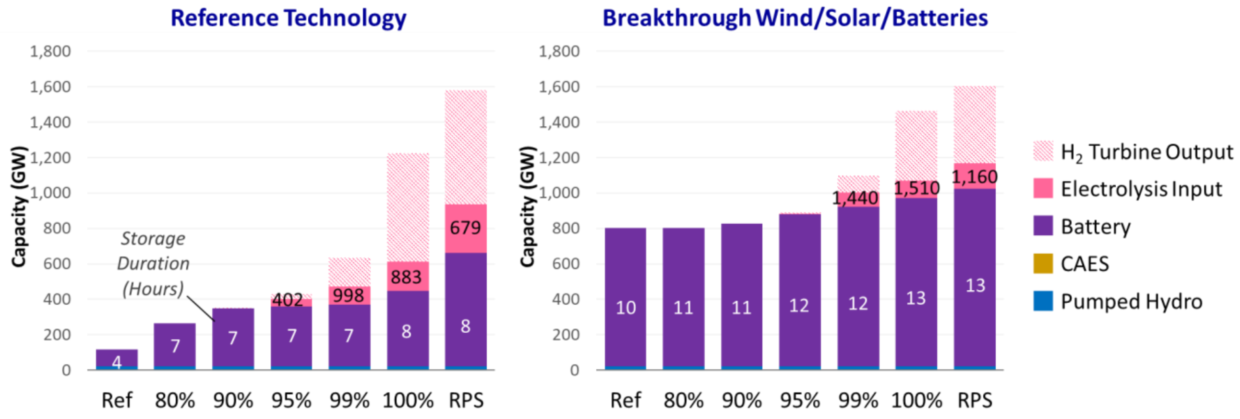

Supplementary Figure 13. Energy storage capacity and duration by technology across different electric sector CO_2 reduction targets (% reduction from 2005 levels). Sensitivities include reference (left) and breakthrough (right) renewables and battery cost assumptions. Scenarios without CDR are shown. Source data are provided as a Source Data file.

Supplementary Figure **14** explores the design space for BECCS and how plausible cost and efficiency assumptions impact BECCS deployment (left) and the CO_2 allowance price (right). BECCS cost and performance assumptions have moderate impacts on deployment, though the variation across a wide parameter range is a small fraction of total installed capacity, as BECCS capacities are between 0 and 41 GW for the sensitivities (national capacity is above 2,000 GW in all scenarios, as shown in Supplementary Figure **8**). Inefficient BECCS could lower abatement costs when carbon removal is more valuable than electricity generation [12], as CO_2 prices are lower with higher BECCS heat rates.

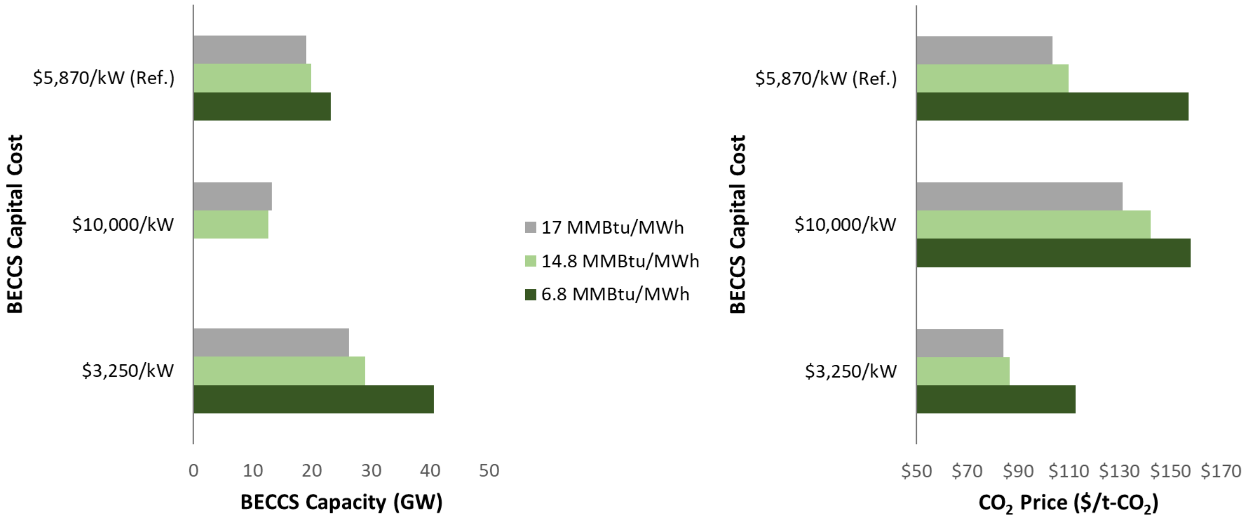

Supplementary Figure 14. BECCS capacity (left) and CO_2 allowance price (right) for 100% electric sector CO_2 cap scenarios (DAC+BECCS). Bars represent sensitivities to the BECCS heat rate and capital costs. Reference cost and heat rate assumptions are based on Johnson and Swisher [5]. Sensitivities come from the literature survey in the recent Energy Modeling Forum 33 study on Bio-Energy and Land Use [13]. Source data are provided as a Source Data file.

Gas capacity factors decline for higher CO_2 reductions, as shown in Supplementary Figure 15. CDR keeps more natural gas capacity online with higher capacity factors. CDR replaces low-utilization, low-CO_2 assets with higher-utilization ones. DAC is only deployed in worlds with high enough CO_2 prices to run with very high capacity factors. BECCS deployment is limited by monthly availability factors but would otherwise run with very high capacity factors.

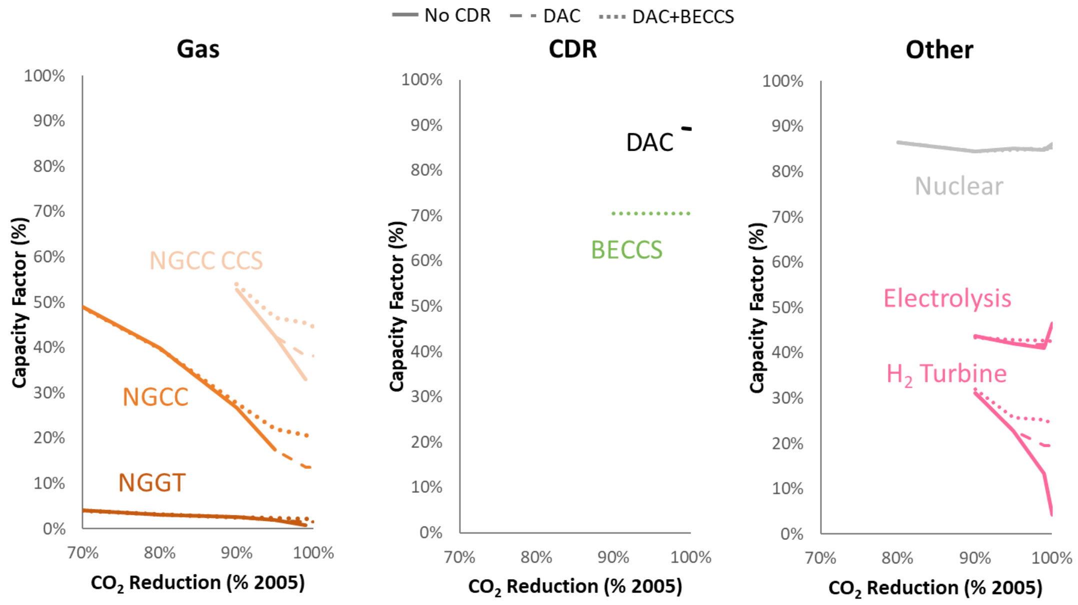

Supplementary Figure 15. Capacity factor by technology and electric sector CO_2 reduction scenario (% reduction from 2005 levels). Lines represent different CDR availability sensitivities. Source data are provided as a Source Data file.

Supplementary Figure 16 shows generation by technology and CO_2 cap scenario for the CDR sensitivities. BECCS deployment slows beginning at 110% reductions, and overall BECCS deployment is a modest but important part of the generation mix to reach net negative emissions goals (123 TWh for the 100% cap, and 260 TWh for the 140% cap). DAC demand leads to relatively small growth (relative to other factors that impact load levels such as electrification, as shown in Figure 7 in the main text) and does not significantly shift generation shares.

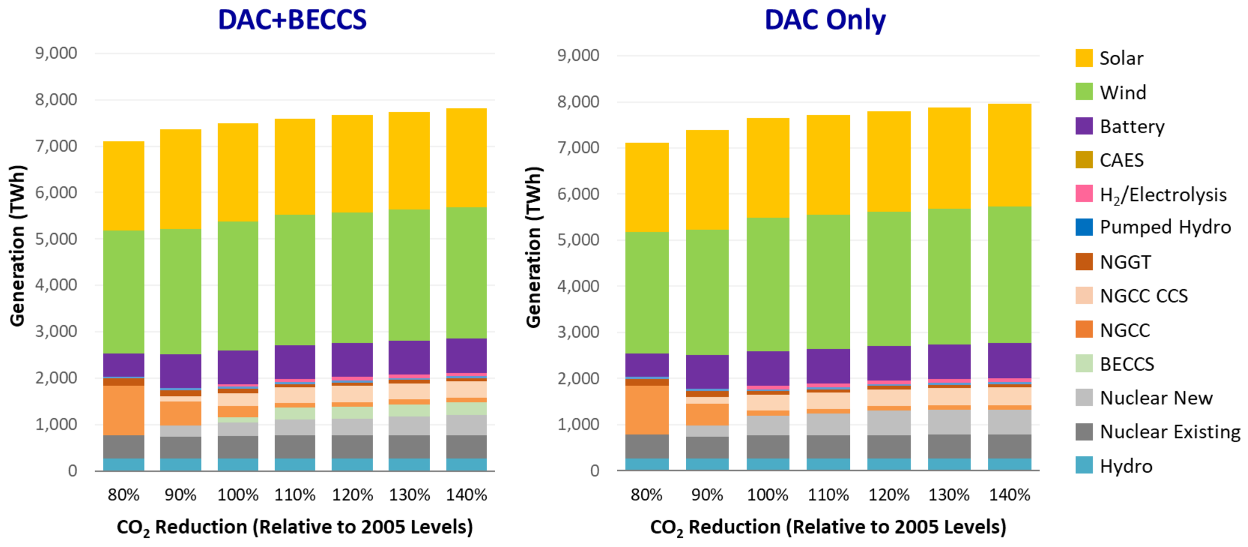

Supplementary Figure 16. National generation by technology and electric sector CO_2 cap scenario (% reduction from 2005 levels). Scenarios with DAC and BECCS (DAC only) shown on the left (right) panel. Source data are provided as a Source Data file.

Supplementary Figure 17 illustrates DAC and BECCS deployment across different levels of policy stringency. With reference costs, BECCS deployment saturates at 110% CO_2 reductions (with a BECCS removal capacity of 436 Mt-CO_2/yr) as marginal biomass feedstock costs increase, and DAC becomes the least-cost CDR technology for additional emissions reductions. This crossover point is reached at 105% (with a BECCS removal capacity of 218 Mt-CO_2/yr) reductions with low biomass resource availability and 90% reductions (with a BECCS removal capacity of 21 Mt-CO_2/yr) with low DAC costs.

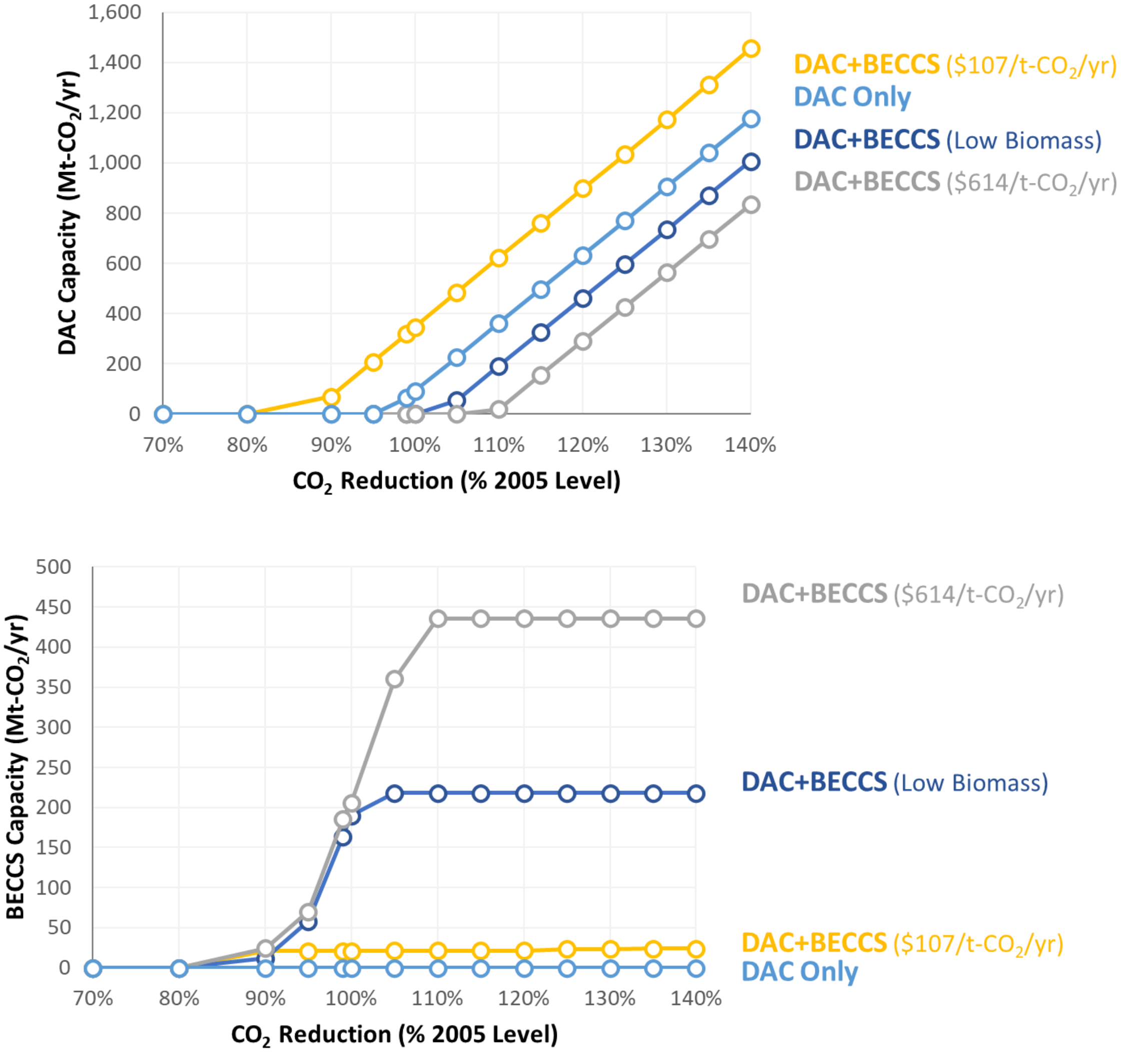

Supplementary Figure 17. CO_2 removal capacity for DAC (top panel) and BECCS (bottom panel) by electric sector CO_2 reduction level (% 2005 levels) and CDR availability scenario. Scenario assumptions are described in Methods. Results are outputs from the REGEN model [4] detailed in Methods and Supplementary Note 1. Source data are provided as a Source Data file.

The levelized cost of net CO_2 removal for BECCS and DAC are compared in Supplementary Figure 18 and Supplementary Figure 19, respectively, for the 140% electric sector CO_2 cap scenario. A key difference is that BECCS produces firm negative-CO_2 electricity generation as a coproduct, though the value of carbon removal is higher than the value of electricity in this example. In equilibrium, the net value of CO_2 removal per ton equals the levelized cost per ton for deployed technologies. For scenarios

and regions where both BECCS and DAC are deployed (e.g., the South Atlantic region below and illustrated in Figure 8 in the main text), the equilibrium levelized costs of net CO_2 removal are equal for BECCS and DAC.

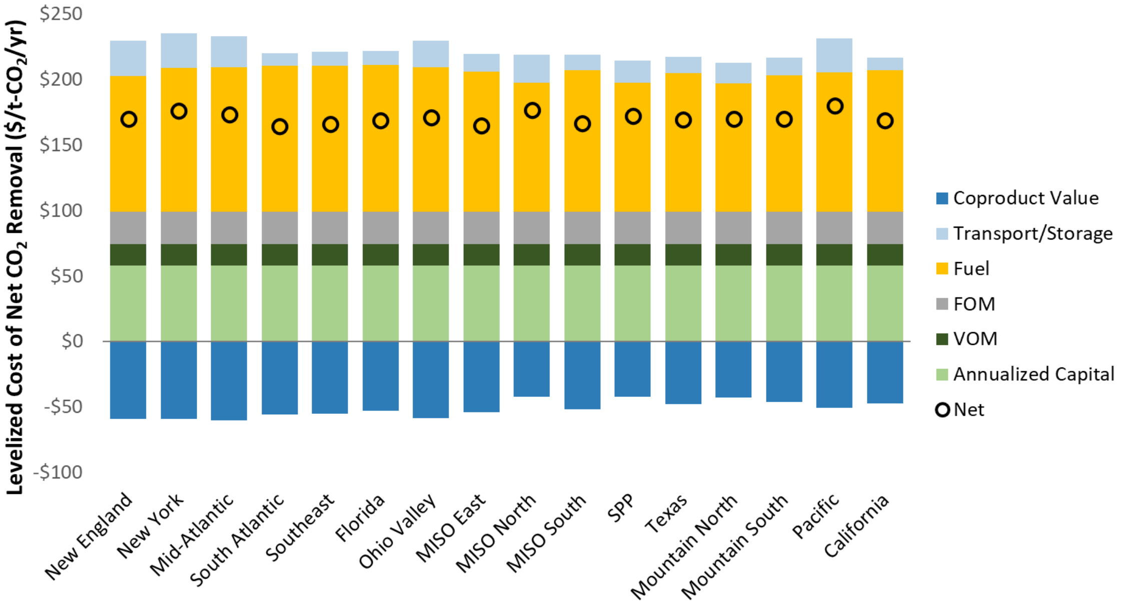

Supplementary Figure 18. Levelized cost of net CO_2 removal (2018 USD per metric ton of net CO_2 captured per year) for BECCS by region. Reference BECCS assumptions are based on Johnson and Swisher [5], which are shown in Table 1 (main text). Values shown for the 140% electric sector CO_2 cap scenario with DAC and BECCS. "Coproduct Value" refers to the electricity produced from BECCS in addition to CO_2 removal. Source data are provided as a Source Data file.

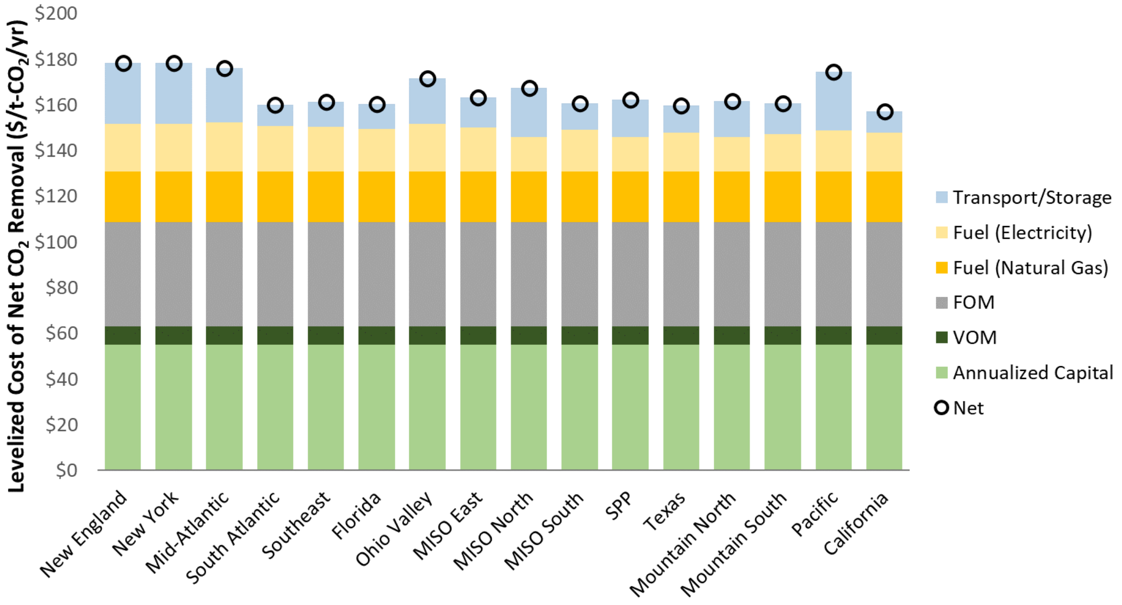

Supplementary Figure 19. Levelized cost of net CO_2 removal (2018 USD per metric ton of net CO_2 captured per year) for DAC by region. DAC assumptions are based on Larsen, et al. [14], which are shown in Table 1 (main text). Values shown for the 140% electric sector CO_2 cap scenario with DAC and BECCS. Source data are provided as a Source Data file.

Biomass supply and use in the 100% and 140% CO_2 reduction scenarios are shown in Supplementary Figure 20.

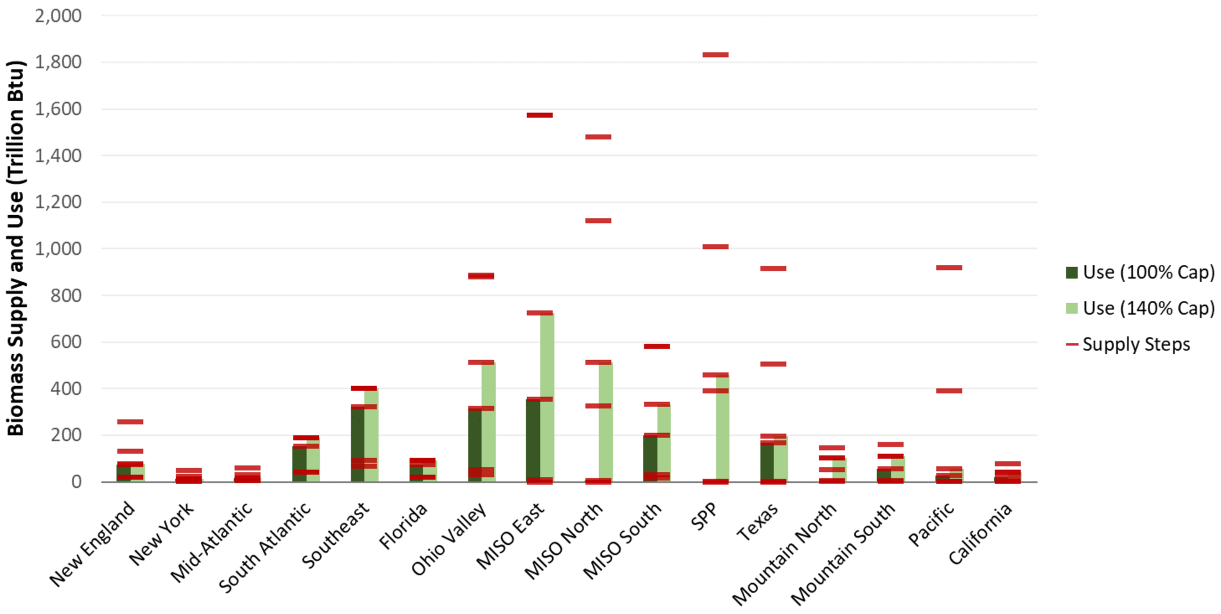

Supplementary Figure 20. Biomass supply and use by region. Values shown for the 100% and 140% electric sector CO_2 cap scenario with DAC and BECCS. Cumulative biomass supply by step in the piecewise linear supply curve (Supplementary Figure **2**) are shown as red dashes. Source data are provided as a Source Data file.

2.2. Economic Impacts

Power sector cost structures shift toward capital expenditures and away from fuel and operating costs, which is a feature of both supply- and demand-side technologies under deeper decarbonization scenarios [15]. Both CO_2 caps and renewable mandates entail capital-intensive systems. CDR availability lowers costs, but having both DAC and BECCS is only slightly lower cost than DAC alone.

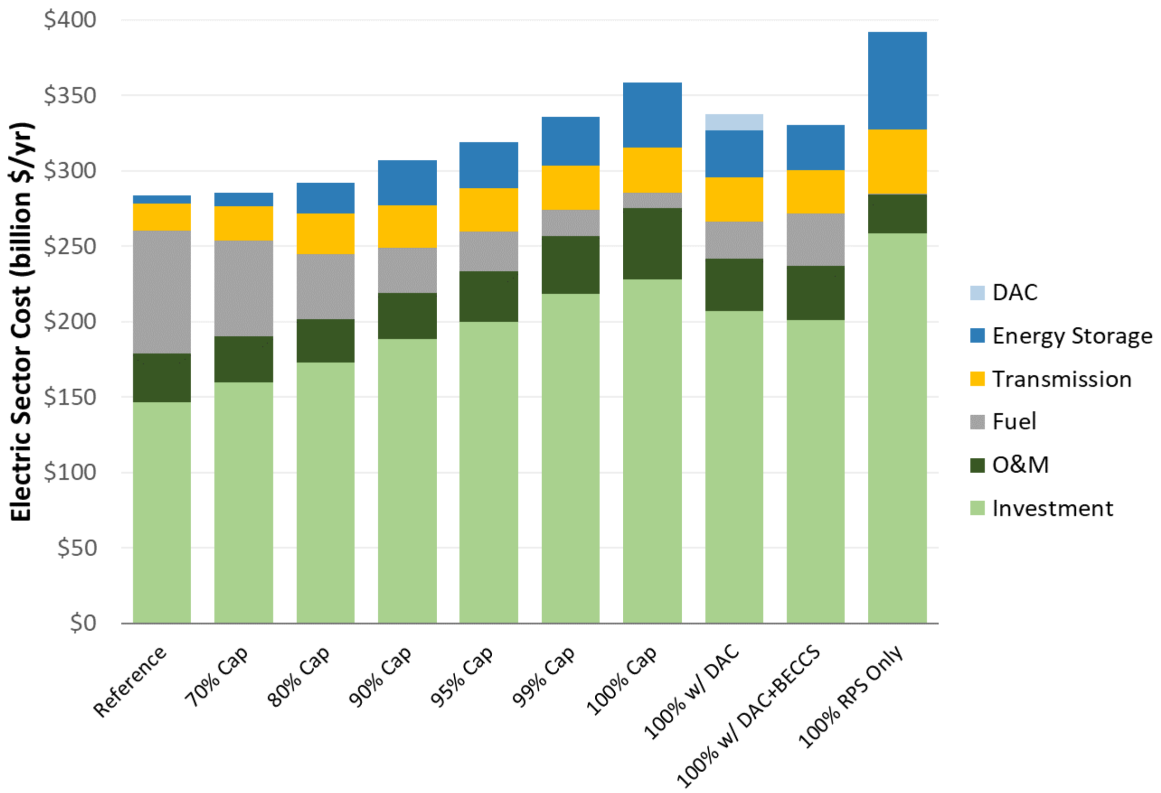

Supplementary Figure 21. Electric sector costs (billion $/yr) by category across decarbonization scenarios and CDR availability sensitivities. "Cap" refers to the electric sector CO_2 cap policy in terms of percentage reductions relative to 2005 levels. Results are based on a seven percent discount rate. Source data are provided as a Source Data file.

CDR lowers the costs of achieving policy goals, but a full benefit-cost assessment requires estimation of non-electric costs offset through CDR. Policy costs increase approximately linearly in abatement owing to DAC flattening cost curve.

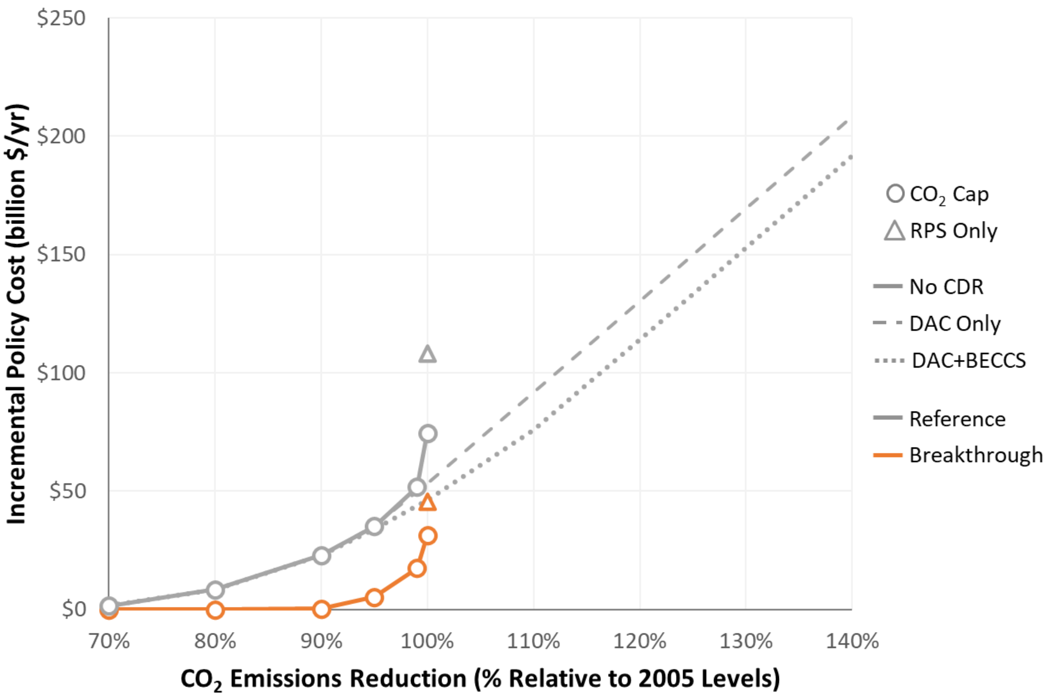

Supplementary Figure 22. Incremental policy cost across different CO_2 reduction targets and assumptions about CDR availability. "RPS Only" limits the choice set of eligible technologies to renewables only. Source data are provided as a Source Data file.

Supplementary Figure 23 illustrates how the availability of CDR technologies can flatten the marginal abatement cost curve by providing a backstop mitigation option. For very high emissions abatement, CDR provides a ceiling on marginal abatement costs, which drop from p_1 to p_2. At the same time, the total cost savings from CDR availability decreases by the red shaded area in Supplementary Figure 23.

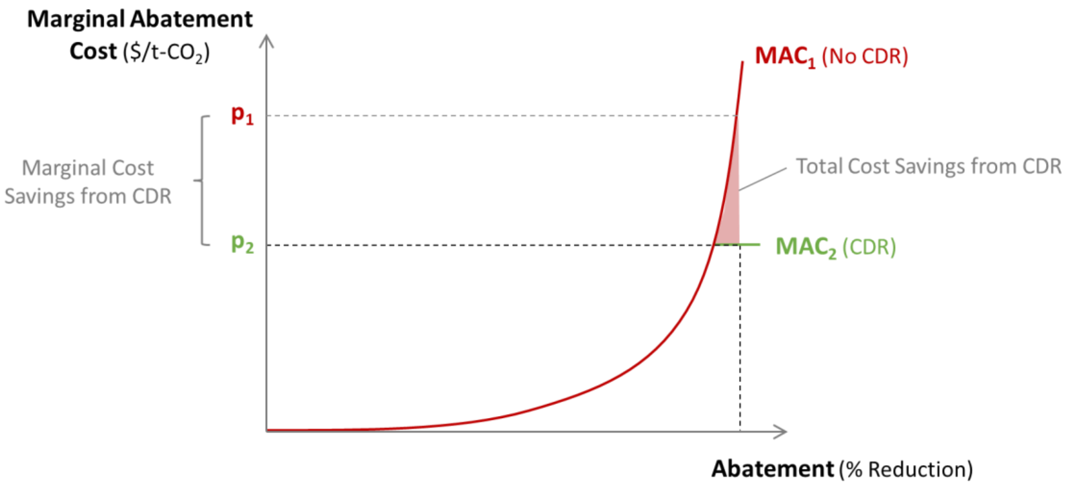

Supplementary Figure 23. Stylized marginal abatement cost curve example. Impacts of carbon dioxide removal (CDR) are shown as the marginal abatement cost curve shifts from MAC_1 (without CDR) to MAC_2 (with CDR), and marginal abatement costs drop from p_1 to p_2.

References

[1] W. Cole, B. Frew, T. Mai, Y. Sun, J. Bistline, G. Blanford, D. Young, C. Marcy, C. Namovicz, R. Edelman, B. Meroney, R. Sims, J. Stenhouse and P. Donohoo-Vallett, "Variable Renewable Energy in Long-Term Planning Models: A Multi-Model Perspective," NREL, Golden, CO, 2017.

[2] S. Collins, P. Deane, K. Poncelet, E. Panos, R. Pietzcker, E. Delarue and B. Gallachóir, "Integrating Short Term Variations of the Power System into Integrated Energy System Models: A Methodological Review," *Renewable and Sustainable Energy Reviews,* vol. 76, pp. 839-856, 2017.

[3] N. Santen, J. Bistline, G. Blanford and F. de la Chesnaye, "Systems Analysis in Electric Power Sector Modeling: A Review of the Recent Literature and Capabilities of Selected Capacity Planning Tools," EPRI, Palo Alto, CA, 2017.

[4] EPRI, "US-REGEN Model Documentation," EPRI, Palo Alto, CA, 2020.

[5] N. Johnson and J. Swisher, "Carbon Capture and Storage in Electric Systems with Restricted Carbon Emissions," EPRI, Palo Alto, CA, 2019.

[6] EPRI, "Solar Plus Storage Cost Assessment and Design Considerations: Executive Summary," EPRI, Palo Alto, CA, 2019.

[7] BNEF, "Hydrogen: The Economics of Production from Renewables," BNEF, New York, NY, 2019.

[8] EPRI, "U.S. National Electrification Assessment," EPRI, Palo Alto, CA, 2018.

[9] J. Bistline, E. Hodson, C. Rossmann, J. Creason, B. Murray and A. Barron, "Electric Sector Policy, Technological Change, and U.S. Emissions Reductions Goals: Results from the EMF 32 Model Intercomparison Project," *Energy Economics,* vol. 73, p. 307–325, 2018.

[10] M. Jafari, M. Korpas and A. Botterud, "Power System Decarbonization: Impacts of Energy Storage Duration and Interannual Renewables Variability," *arXiv preprint,* p. 12331, 2019.

[11] P. Albertus, J. Manser and S. Litzelman, "Long-Duration Electricity Storage Applications, Economics, and Technologies," *Joule,* 2019.

[12] N. Mac Dowell and M. Fajardy, "Inefficient Power Generation as an Optimal Route to Negative Emissions via BECCS?," *Environmental Research Letters,* vol. 12, no. 4, p. 045004, 2017.

[13] V. Daioglou, S. Rose, N. Bauer, A. Kitous, M. Muratori, F. Sano, S. Fujimori, M. Gidden, E. Kato, K. Keramidas and D. Klein, "Bioenergy Technologies in Long-Run Climate Change Mitigation: Results from the EMF-33 Study," *Climatic Change,* vol. 163, no. 3, pp. 1603-1620, 2020.

[14] J. Larsen, W. Herndon, M. Grant and P. Marsters, "Capturing Leadership: Policies for the US to Advance Direct Air Capture Technology," Rhodium, New York, NY, 2019.

[15] E. Larson, C. Greig, J. Jenkins, E. Mayfield, A. Pascale, C. Zhang, J. Drossman, R. Williams, S. Pacala and R. Socolow, "Net-Zero America: Potential Pathways, Infrastructure, and Impacts," Princeton University, Princeton, NJ, 2020.
